# Application of a Limit-Cycle Oscillator Model for Prediction of Circadian Phase in Rotating Night Shift Workers

**DOI:** 10.1038/s41598-019-47290-6

**Published:** 2019-07-30

**Authors:** Julia E. Stone, Xavier L. Aubert, Henning Maass, Andrew J. K. Phillips, Michelle Magee, Mark E. Howard, Steven W. Lockley, Shantha M. W. Rajaratnam, Tracey L. Sletten

**Affiliations:** 1 CRC for Alertness, Safety and Productivity, Melbourne, Victoria Australia; 2 School of Psychological Sciences and Turner Institute for Brain and Mental Health, Faculty of Medicine, Nursing & Health Sciences, Monash University, Clayton, Victoria Australia; 3 Philips Research, Eindhoven, The Netherlands; 4 Institute for Breathing and Sleep, Austin Health, Victoria, Australia; 5 Division of Sleep and Circadian Disorders, Departments of Medicine and Neurology, Brigham and Women’s Hospital, Boston, Massachusetts USA; 6 Division of Sleep Medicine, Harvard Medical School, Boston, Massachusetts USA

**Keywords:** Oscillators, Circadian rhythms and sleep, Computational models

## Abstract

Practical alternatives to gold-standard measures of circadian timing in shift workers are needed. We assessed the feasibility of applying a limit-cycle oscillator model of the human circadian pacemaker to estimate circadian phase in 25 nursing and medical staff in a field setting during a transition from day/evening shifts (diurnal schedule) to 3–5 consecutive night shifts (night schedule). Ambulatory measurements of light and activity recorded with wrist actigraphs were used as inputs into the model. Model estimations were compared to urinary 6-sulphatoxymelatonin (aMT6s) acrophase measured on the diurnal schedule and last consecutive night shift. The model predicted aMT6s acrophase with an absolute mean error of 0.69 h on the diurnal schedule (SD = 0.94 h, 80% within ±1 hour), and 0.95 h on the night schedule (SD = 1.24 h, 68% within ±1 hour). The aMT6s phase shift from diurnal to night schedule was predicted to within ±1 hour in 56% of individuals. Our findings indicate the model can be generalized to a shift work setting, although prediction of inter-individual variability in circadian phase shift during night shifts was limited. This study provides the basis for further adaptation and validation of models for predicting circadian phase in rotating shift workers.

## Introduction

Working and sleeping out of phase with the endogenous circadian pacemaker is a major factor contributing to adverse health and safety outcomes^1,2^. Biological adaptation of the circadian clock to a night shift schedule, marked by a shift in the timing of melatonin secretion, into the daytime rest period, can improve sleep quality and reduce performance impairments^3–5^. Even partial circadian adaptation is, however, rarely observed in most real-world settings^6^.

Multiple field studies have reported large variability between individuals in the direction and magnitude of circadian phase shift in response to night shift work^7–10^. This inter-individual variability has been linked to differences in the timing of light exposure^7,9,11^, which is the key timing signal for the circadian clock^12–14^. Night workers with delayed circadian phase after consecutive night shifts are exposed to more light during the evening compared to those with advanced phase^7^. Inter-individual variability in the magnitude and direction of circadian phase response to night shift can be largely explained by the relative difference in the amount of light exposure in the delay and advance portions of the phase response curve^11^.

Individual differences in circadian response to night work translate to variation between workers in the time course of alertness and performance across the night shift^5,10,15–17^. Knowledge of individual circadian phase in shift workers could identify times of impaired alertness and thereby inform individualized countermeasures for improving workplace safety, overall health, and wellbeing. Direct assessment of circadian phase via measurement of the melatonin rhythm is impractical and prohibitively expensive for ongoing use in occupational settings. Alternative, non-invasive methods to measure or estimate circadian rhythms in shift workers are therefore needed.

Mathematical modeling based on ambient light exposure may be a promising method for the prediction of individual circadian phase in night workers. A previously developed model of the human circadian pacemaker predicts the response of the pacemaker to environmental stimuli, particularly light-dark exposure^18,19^. The model simulates the regular oscillations of the circadian pacemaker, which is represented as a limit-cycle oscillator, and models the ability of light stimuli to move the oscillator away from the limit cycle. The pace and amplitude of the oscillations is thereby altered, resulting in changes in the predicted phase of the pacemaker rhythm. The timing and strength of the light stimulus modulate the effect of light on the pacemaker, based on experimental findings on the human phase response curve to light, and dose response curves tested in controlled laboratory settings^19–24^. An additional non-photic component, based on the timing of activity-rest patterns, has been added to allow for potential non-light effects on the circadian pacemaker^19^.

While the model has been validated on experimental data^18,19,25^, less is known about the accuracy of model predictions in a field setting, especially in shift worker populations. Prior implementations using light and activity data collected under normal living conditions on a diurnal sleep-wake schedule^26–29^ indicate the model may be used to predict circadian phase in the field with an average accuracy of approximately ±1 hour. Furthermore, findings in healthy participants indicate that the addition of activity information, implemented either as the peak of the daily activity rhythm^27^, or as a non-photic component of the limit-cycle oscillator model^26^, may improve circadian phase predictions when using field data.

Whether the model can reliably estimate circadian phase in a real-world shift work population has not yet been evaluated. This study aimed to assess the feasibility of using the model to predict circadian phase response to a night shift schedule using ambulatory light and activity data in rotating shift workers, working a transition from diurnal shift schedule (day/evening shifts and days off) to multiple consecutive night shifts (3–5 nights).

## Results

Characteristics of the 25 participating doctors and nurses are summarized in Table 1. Activity and light data, measured using wrist-worn actigraphs, were available for 7.16 ± 3.01 (M ± SD; range 2–14) consecutive 24-h intervals over a work schedule consisting of day/evening shifts and days off (the *diurnal schedule*), and 3.60 ± 1.08 (M ± SD; range 3–5) consecutive 24-h intervals over a schedule consisting of 3–5 consecutive night shifts (the *night schedule*). The number of days of actigraphy data (light and activity counts) available varied between participants depending on when they were enrolled into the study relative to their night schedule.

**Table 1:** Participant characteristics.

	n	Mean	Min	Max	Mean	Min	Max
Number (male, female)	25 (7, 18)						
Occupation (nurse, doctor)	25 (20, 5)						
Age (years)		33.32 (9.19)	24	58			
MEQ score		38.32 (5.70)	28	52			
BMI (kg/m^2^)		23.84 (3.70)	15.9	29.9			
No. days of data prior to diurnal acrophase		7.20 (2.89)	2	14			
No. night shifts		3.76 (0.72)	3	5			
		**Diurnal Schedule**			**Night Schedule**		
Average mid-sleep (h)		03:19 (0:36)	02:35	04:51	12:38 (01:08)	10:48	15:00
Average bed time (h)		23:16 (0:13)	22:14	00:26	9:20 (00:58)	07:54	11:52
Average wake time (h)		07:23 (0:53)	06:15	09:17	15:57 (01:30)	13:10	18:40
Average rest duration (h)		08:07 (0:48)	06:42	09:48	06:39 (01:07)	03:48	08:24

Note: MEQ = Morningness-Eveningness Composite Questionnaire, BMI = Body Mass Index, aMT6s = urinary 6-sulphatoxymelatonin, SD = standard deviation.

### Measured circadian phase results

**Figure 1:**
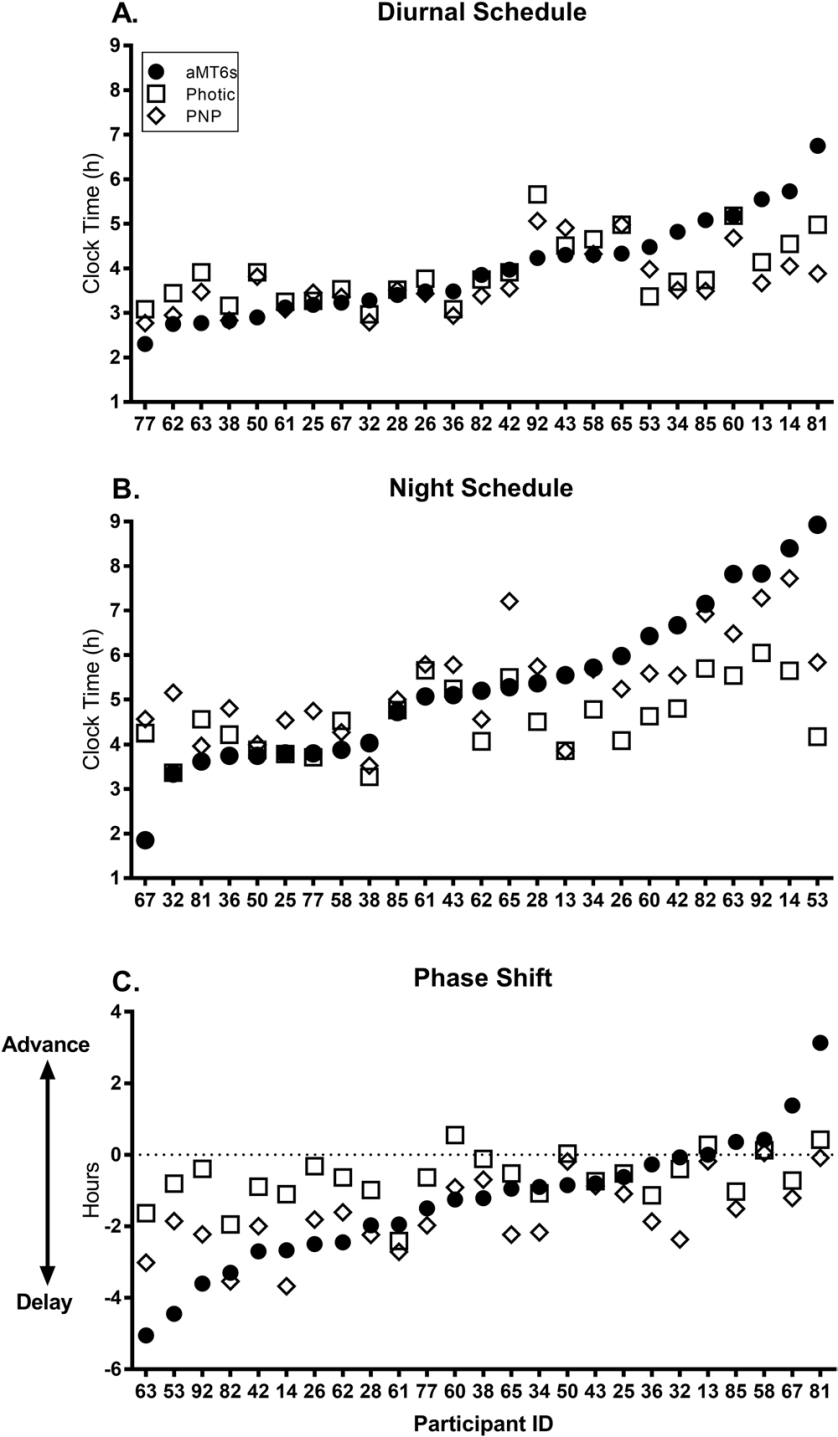
Measured aMT6s acrophase ranked from earliest to latest clock time, with predicted acrophase using photic and PNP models, for each subject. (**A**) Acrophase times on diurnal schedule for n = 25 participants. (**B**) Acrophase times on third, fourth or fifth consecutive night shift for n = 25 participants. (**C**) Phase shift from diurnal schedule to final night shift for n = 25 participants. Positive phase shift indicates a phase advance (i.e., final night shift phase occurring at an earlier clock time than diurnal phase); negative phase shift indicates a phase delay (i.e., final night shift phase occurring at a later clock time than diurnal phase). Circles represent measured aMT6s acrophase time (reference phase); squares represent predicted phase using the photic only mode; diamonds represent predicted phase using the photic and non-photic (PNP) model.

The acrophase (peak) in the urinary 6-sulphatoxymelatonin (aMT6s) rhythm was used as a marker of circadian phase. On the diurnal schedule, aMT6s acrophase occurred at 3:58 ± 1:06 h (M ± SD; range 2:18–6:45 h; Fig. 1A). After 3 to 5 consecutive night shifts, aMT6s acrophase occurred at 5:19 ± 1:45 h (M ± SD; range 1:51–8:55 h; Fig. 1B), representing an average phase delay of 1:21 ± 1:48 h (M ± SD; range 5:03 h delay to 3:07 h advance). Over the final night shift, the majority of participants experienced a phase delay (n = 21), with only 4 participants experiencing a phase advance (Fig. 1C).

### Model estimations of circadian phase

#### Photic only model

Using only the photic input, ${\rm{B}}({\rm{t}})$, the model estimated an average aMT6s acrophase for all participants on the diurnal schedule of 3:55 ± 0:43 h (range: 2:58–5:39 h). After 3 to 5 consecutive night shifts the photic model predicted an average aMT6s acrophase of 4:34 ± 0:46 h (range: 3:16–6:03 h). The average predicted phase shift was a delay of 0:39 ± 0:42 h (range 2:24 h phase delay to 0:33 h phase advance). The photic-only model accurately predicted aMT6s acrophase to within ±30 minutes in 52% and 32% of individuals, and to within ±1 hour in 64% and 56% on the diurnal and night schedules, respectively (Table 2). The direction of phase shift was predicted accurately in 80% of participants (12% incorrect prediction of phase advance; 8% incorrect prediction of phase delay). The model predicted a total of 5 phase advances, of which only 2 accurately reflected the direction of aMT6s phase shift.

**Table 2:** Summary of prediction error in hours for photic only and PNP models across the shift schedule.

	n	Mean (h)	Range (h)	Prediction Error Mean (h)	Prediction Error Absolute Mean (h)	Prediction Error Min (h)	Prediction Error Max (h)	Prediction within ±30 min	Prediction within ±60 min	Prediction within ±120 min
***Diurnal Schedule***										
aMT6s Phase	25	3.97 (1.10)	2.30–6.75							
Photic Only Model	25	3.92 (0.73)	2.97–5.66	0.05 (0.85)*	0.65 (0.53)	0.00	1.77	52%	**64%**	100%
PNP Model	25	3.67 (0.68)	2.77–5.06	0.30 (0.94)	0.69 (0.69)	0.01	2.87	56%	**80%**	96%
***Night Schedule***										
aMT6s Phase	25	5.32 (1.76)	1.85–8.93							
Photic Only Model	25	4.58 (0.78)	3.28–6.05	0.74 (1.46)**	1.19 (1.11)	0.01	4.75	32%	**56%**	84%
PNP Model	25	5.35 (1.13)	3.53–7.73	−0.03 (1.24)	0.95 (0.77)	0.04	3.09	28%	**68%**	92%
***Phase Shift***										
aMT6s Phase Shift	25	−1.35 (1.81)	−5.05–3.13							
Photic Only Model	25	−0.66 (0.70)	−2.41–0.56	−0.69 (1.60)**	1.30 (1.08)	0.00	3.65	32%	**48%**	76%
PNP Model	25	−1.68 (1.02)	−3.68–0.06	0.33 (1.39)	1.11 (0.88)	0.07	3.22	32%	**56%**	80%

Note: aMT6s = urinary 6-sulphatoxymelatonin, PNP = photic and non-photic inputs, SD = standard deviation, *p < 0.05; **p < 0.001 for comparisons between photic and PNP models.

**Figure 2:**
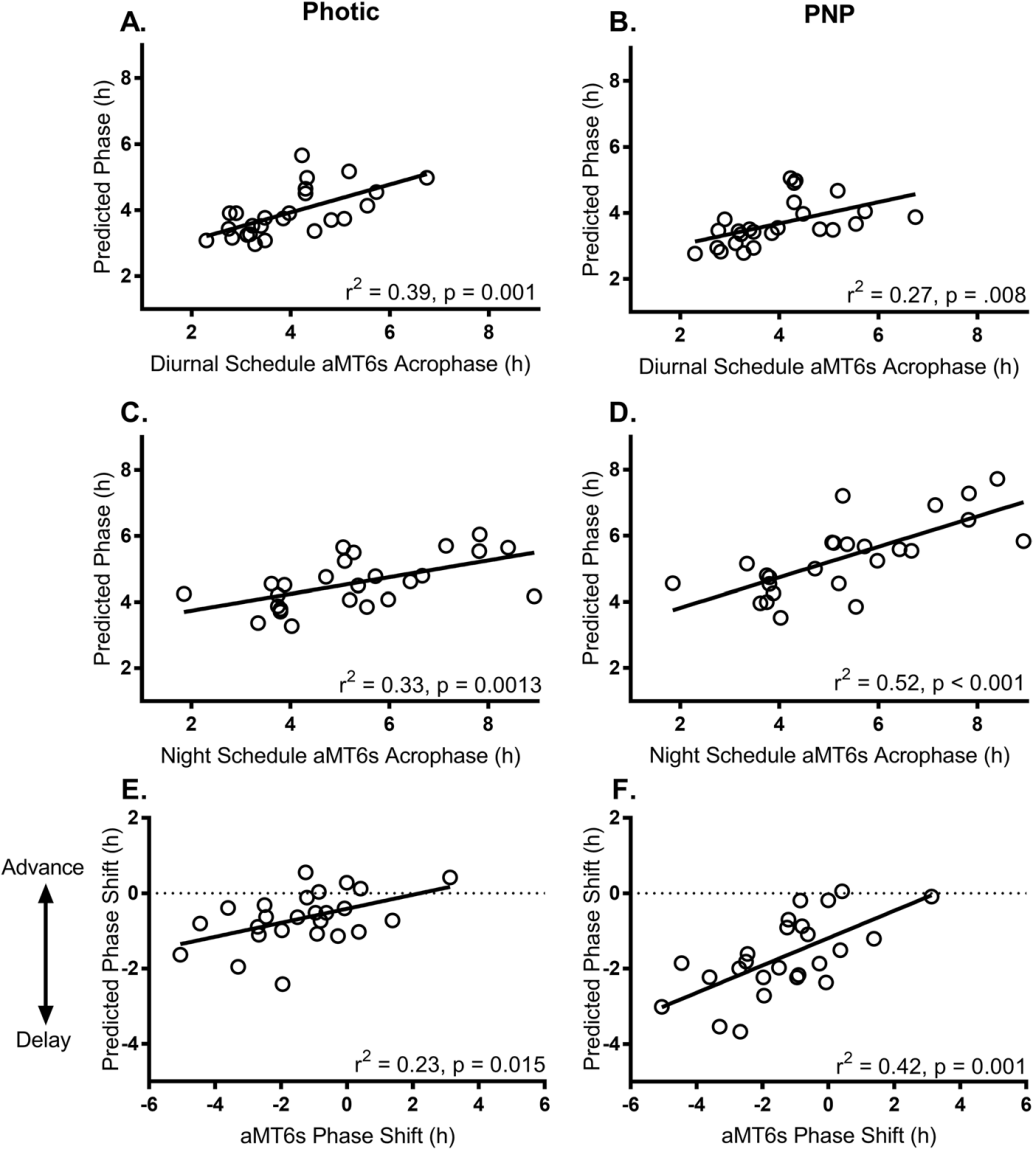
Relationship between acrophase estimated using the photic-only model (left panels) and the photic and non-photic (PNP) model (right panels), and measured aMT6s acrophase on diurnal schedules (**A**,**B**), after 3 to 5 consecutive night shifts (**C**,**D**), and phase shift between day and night shifts (**E**,**F**). Positive phase shift indicates a phase advance (i.e., final night shift acrophase earlier than diurnal acrophase); negative phase shift indicates a phase delay (i.e., final night shift phase later than diurnal acrophase).

Model phase estimates generated using the photic model had a significant positive relationship with measured aMT6s acrophase on the diurnal schedule (r = 0.63, r^2^ = 0.39, p = 0.001; Fig. 2A), and on final night shift (r = 0.60, r^2^ = 0.33, p = 0.001; Fig. 2C), and with the degree of phase shift (r = 0.48, r^2^ = 0.23, p = 0.015; Fig. 2E). Thus, 39% and 33% of the variance in aMT6s acrophase was explained by the photic model on the diurnal schedule and final night shift, respectively, and 23% of the variance in aMT6s phase shift. At the group level, predictions of phase on the diurnal schedule were not significantly different from aMT6s acrophase times (t(24) = 0.30, p = 0.768). Predicted phase on final night shift, however, was significantly different to measured aMT6s acrophase times (t(24) = 2.52, p = 0.019), indicating an under-prediction of the delay in aMT6s acrophase times. Correspondingly, predicted phase shift differed significantly from measured shift in aMT6s acrophase over the night shift schedule (t(24) = −2.15, p = 0.04), indicating an under-prediction of the magnitude of phase shifts, particularly when there were phase shifts of 2 hours or more.

#### Photic and non-photic (PNP) model

With the additional non-photic term (photic and non-photic model; PNP), ${\rm{N}}({\rm{t}})$, the model predicted an average aMT6s acrophase on the diurnal schedule of 3:40 ± 0:40 h (range: 2:46–5:03 h). After 3 to 5 consecutive night shifts, the PNP model predicted an average aMT6s acrophase of 5:21 ± 1:07 h (range: 3:31–7:43 h). The PNP model accurately predicted aMT6s acrophase to within ±30 minutes in 56% and 28% of individuals, and to within ±1 hour in 80% and 68% of individuals on the diurnal and night schedules, respectively (Table 2). The predicted phase shift based on PNP model predictions was an average phase delay of 1:40 ± 1:01 h (range: 3:40 h phase delay to 0:03 h phase advance). The PNP model correctly predicted the direction of phase shift in 88% of participants (12% incorrect prediction of phase delay). Only one phase advance was predicted, which accurately reflected the direction of the measured aMT6s phase shift for that participant.

Model-predicted phase using the PNP inputs was significantly associated with measured aMT6s acrophase on the diurnal schedule (r = 0.52, r^2^ = 0.27, p = 0.008; Fig. 2B), on final night shift (r = 0.72, r^2^ = 0.52, p < 0.001; Fig. 2D), and phase shift (r = 0.65, r^2^ = 0.42, p = 0.001; Fig. 2F). Thus, 27% and 52% of the variance in aMT6s acrophase was explained by the PNP model on the diurnal schedule and final night shift, respectively, and 42% of the variance in aMT6s phase shift. At the group level, model predictions did not differ significantly from measured mean aMT6s acrophase on the diurnal schedule (t(24) = 1.58, p = 0.128), on final night shift (t(24) = −0.13, p = 0.901), or the aMT6s phase shift (t(24) = 1.19, p = 0.250).

#### Prediction error relative to measured aMT6s acrophase

**Figure 3:**
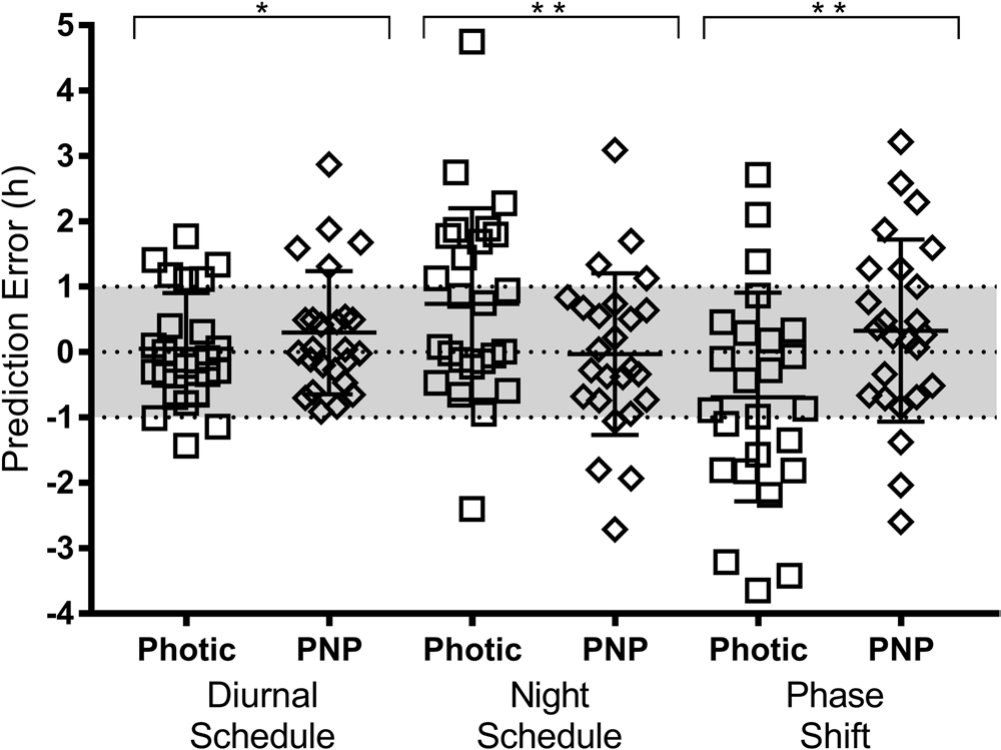
Prediction error in hours for diurnal acrophases, final night shift acrophases, and phase shifts. Prediction error is calculated as measured aMT6s acrophase minus predicted phase. Square symbols represent prediction error from the photic model; diamond symbols represent prediction error from the PNP model predictions. Mean and standard deviation for each model are shown as line and error bars. Prediction error of ±1 hour is shaded. *p < 0.05; **p < 0.001 for comparisons between photic and PNP models.

The prediction error for each model is summarized in Table 2. Model performance using the photic-only and PNP models were comparable when predicting diurnal phase. On the night shift schedule the PNP model showed slightly improved performance compared to the photic-only model, across all metrics of prediction error. There were no significant differences in mean absolute prediction error between photic and PNP models on either diurnal (p = 0.66) or night shift (p = 0.23) schedules, or in phase shift (p = 0.21). When comparing raw error values mean prediction error was smaller when predicting diurnal phase using the photic only model compared to the PNP model (t(24) = −3.678, p = 0.001; Fig. 3). Conversely, mean prediction error was smaller when using the PNP model to predict phase on final night shift (t(24) = 5.757, p < 0.001), or to predict phase shift between the shift schedules (t(24) = −7.889, p < 0.001). There was an offset in mean prediction error (raw error values) using the photic model to predict acrophase on the night schedule such that predicted phase was systematically earlier than aMT6s acrophase.

**Figure 4:**
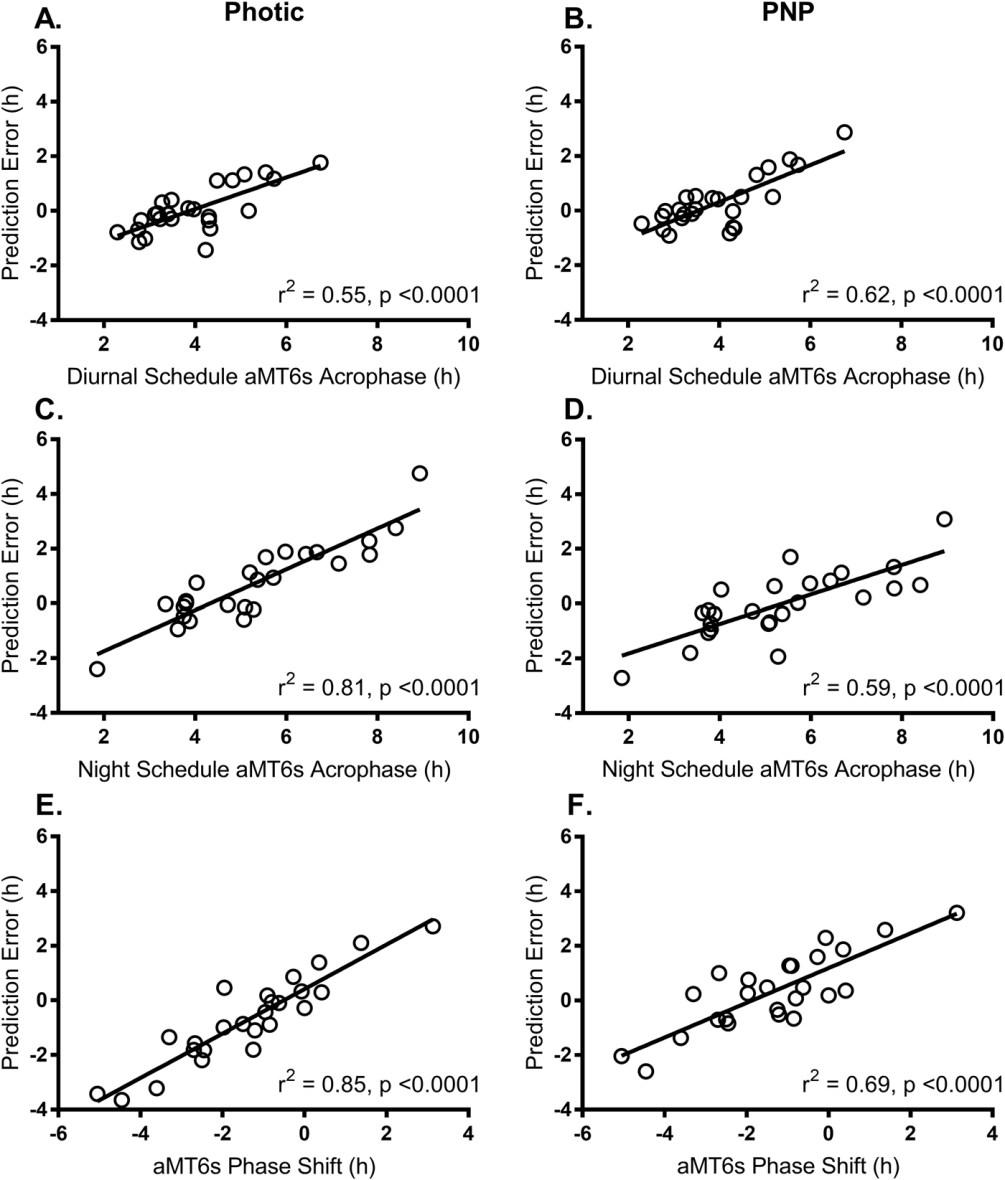
Relationship between prediction error and measured aMT6s acrophase measured on the diurnal schedule, night schedule and shift in phase between schedules, for photic model predictions (left panel) and PNP model predictions (right panel).

For both models, there were significant relationships between prediction error and (1) the measured diurnal acrophase, (2) final night shift acrophase, and (3) phase shift (Fig. 4). This relationship was strongest when using the photic model to estimate acrophase on final night shift (r^2^ = 0.81, p < 0.001; Fig. 4C) or phase shift from day to final night shift (r^2^ = 0.85, p < 0.001; Fig. 4E). There were no significant relationships between prediction error (for predicted diurnal or night phase, or predicted phase shift) and age, BMI, average mid-sleep time on the diurnal schedule, or MEQ score.

#### Removal of outlier on diurnal schedule

One participant (81) had a relatively late aMT6s acrophase time on the diurnal schedule (6:45 am; more than 2 SD from the mean of 3:58 am ± 1:06 h), with large prediction error when using both photic and PNP models. To examine a potential bias caused by this participant, analyses were also conducted with this participant removed. This resulted in a smaller average prediction error for the photic model (absolute mean = 0.61 ± 0.49 h), but did not improve the strength of the relationship between predicted phase and measured aMT6s acrophase (r^2^ = 0.34, p = 0.003). There was also a reduction in prediction error for the PNP model (absolute mean = 0.60 ± 0.54 h), with an improved relationship between predicted and measured phase (r^2^ = 0.33, p = 0.003).

#### Impact of initial values on model estimations

To test whether model performance for predicting final night shift phase could be improved with the application of individual starting acrophase, the models were initialized using diurnal aMT6s acrophase (the “oracle” method). Initializing with the known initial acrophase made only a small improvement in the predictions of acrophase on final night shift, and in the predictions of phase shift (see Table S1). When applying the “oracle” model initialization using the PNP model, the mean absolute prediction error in the night schedule reduced from 0.95 h to 0.94 h, and the mean absolute prediction error in the phase shift reduced from 1.11 h to 1.04 h. Model predictions using the PNP model improved from accounting for 52% to 53% of variance in aMT6s night shift acrophase; and from 42% to 49% of variance in phase shift (Fig. S2).

## Discussion

This study tested the capability of a mathematical limit-cycle oscillator model to predict circadian timing in the context of rotating night shift work, using easily collected ambulatory light and activity data. The main findings are: (1) the photic and PNP models both performed well on diurnal schedules, with absolute mean prediction errors <0.7 h, and >50% of aMT6s acrophases predicted to within ±30 minutes; (2) the addition of a non-photic input (i.e., incorporating the effects of sleep/wake patterns on circadian phase) improved prediction of aMT6s acrophase after night shifts from an absolute mean error of 1.19 h to 0.95 h; (3) predicted phase shifts were of smaller magnitude than the measured aMT6s phase shifts; (4) knowledge of aMT6s acrophase prior to night shifts only modestly improved model phase estimations compared to initializing with a starting point derived from mid-sleep time.

The photic and PNP models that we tested here have been extensively developed from measurements of human circadian responses under laboratory conditions in highly-screened, healthy individuals^18,19,25^. Although these models have been used to assess real-world schedules, including rotating night shift schedules^30^, prediction accuracy under such conditions had not been previously assessed. Recent field studies of individuals living on diurnal schedules demonstrated that these models can predict timing of melatonin phase markers to within ±1 hour in about half of individuals using wrist-worn light and activity data^26–28^. We replicated these findings and for the first time evaluated prediction accuracy on night schedules, including considerable variations in individual shift rosters. We conclude that the PNP model is a suitable tool for predicting phase on night schedules, albeit with slightly less accuracy than on diurnal schedules. The importance of including non-photic inputs is an important finding for operational use, and likely reflects the fact that abnormal phase angles between sleep timing and circadian timing often arise on rotating shift schedules. Specifically, we observed improved prediction of later circadian phases, potentially due to the impact of the delayed sleep-wake timing during the night schedule on model accuracy.

**Figure 5:**
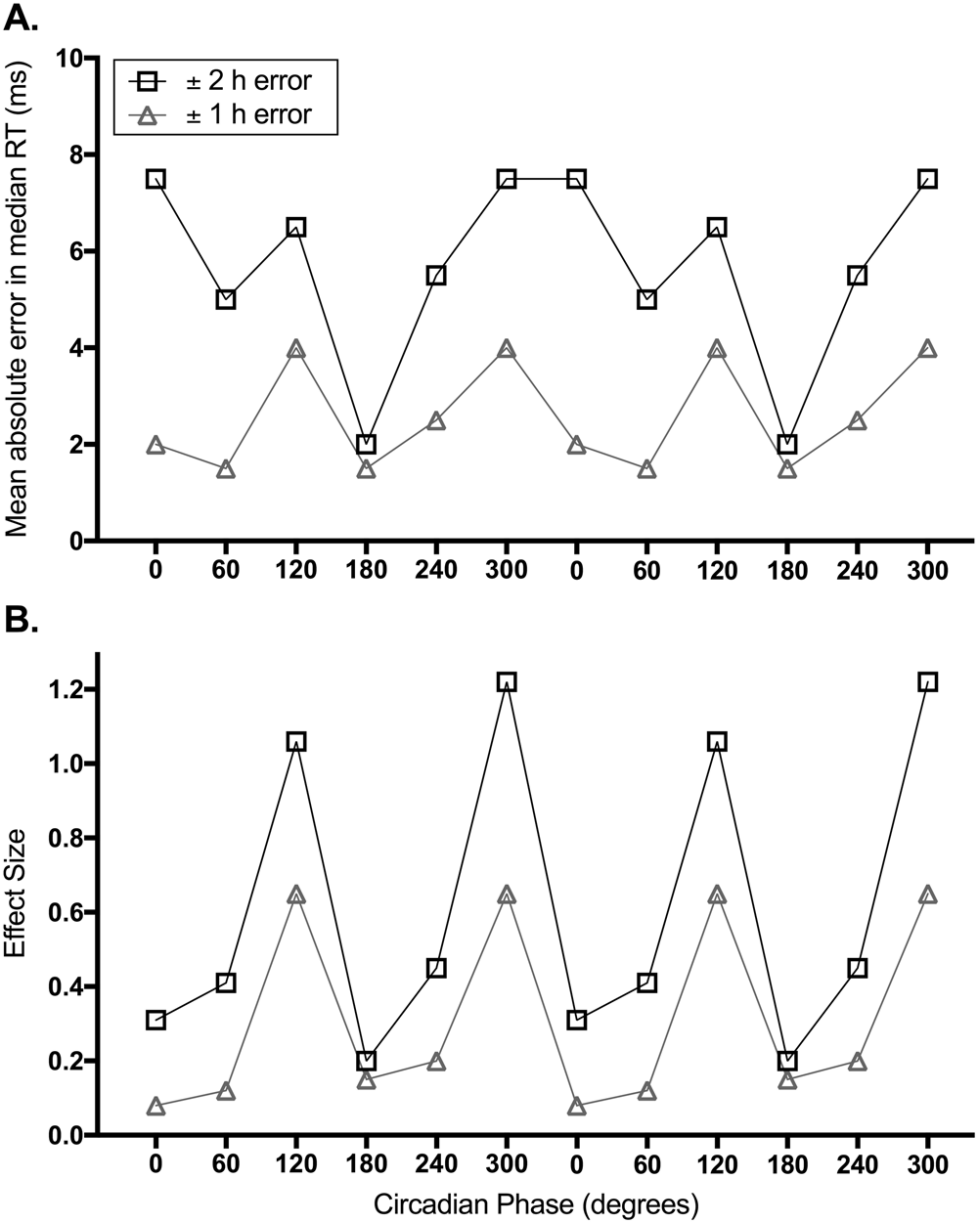
Impact of a one-hour versus two-hour error in circadian phase estimation on psychomotor performance reaction time, calculated using published forced desynchrony data^31^. (**A**) Mean absolute difference in median reaction time (ms) with a ± 1 hour error (grey triangles) and a ± 2 hour error (black squares) in circadian phase, across the biological day. (**B**) Effect size of median reaction time error with a ± 1 hour error (grey triangles) and a ± 2 hour error (black squares) in circadian phase, across the biological day. Circadian phase is double plotted and represented in degrees, where 0 = CBTmin.

Whenever predicting circadian phase, an important but rarely addressed question is: what level of accuracy is required? For example, is a 1 hour error tolerable? The answer will naturally depend upon the outcome being predicted. A key application of predictive models in shift work settings is determining times of likely poor cognitive performance (e.g.,^30^). We can estimate the consequences of different circadian phase prediction errors from laboratory data. Using median reaction time on a psychomotor vigilance task (PVT) under forced desynchrony conditions^31^, we calculated the absolute errors in reaction time and their effect sizes for a 1 or 2 hour error in phase (see Fig. 5). This analysis indicates that effect size varies by circadian phase (i.e., the consequences of a mis-estimate are greater at some circadian phases than others), and that a 1 hour error ensures a medium or smaller effect size, whereas a 2 hour error can result in large effect sizes. These findings suggest that, at least for a cognitive performance outcome, the mean absolute error of 0.95 h obtained for the PNP model under night shift conditions is tolerable. We note, however, that other, non-circadian factors could contribute to prediction errors for cognitive performance, such as the sleep homeostatic process.

Our findings identify areas of potential improvement for the models. First, the models did not replicate the amount of inter-individual variability in circadian phase observed, either on diurnal or night shift schedules. This discrepancy is indicated by the regression lines for predicted versus actual phase which have slopes consistently <1, similar to previous findings^28^. This is unsurprising given that the model uses the same parameter values for all individuals, whereas we know there are individual differences in circadian physiology, including intrinsic circadian period^12,32^ and individual light sensitivity^33,34–37^. Tuning the τ_c_ parameter for males and females based on published sex differences in circadian period^12^ did not improve model performance in this sample. This issue could potentially be addressed by the development of tools to individualize estimation of model parameters^38–40^. We did not attempt to perform model parameter individualization on this dataset, since we did not have direct measures of τ_c_ or other model parameters. Second, the models systematically underestimated the amount of phase delay that occurred, particularly for individuals with phase shifts of 2 hours or more. This finding is consistent with a previous application of the model, where the model underestimated phase shifts over a simulated night shift protocol^41^.

A possible reason for the underestimation of phase shifts is limited validation of the model in real-world environments with low light levels such as during night shifts. Participants in this study were exposed to lower average intensity light, and spent a smaller proportion of time in bright light whilst working night shifts, compared to on the diurnal schedule. Participants on night shift spent 24% of waking hours at <10 lux and 61% of waking hours at <100 lux. Laboratory experiments have demonstrated a nonlinear relationship between light intensity and circadian phase response, with half the maximal phase resetting observed at ~100 lux^42^. While the light parameters in the model have been refined to reflect these studies^19^, our findings suggest that the model may still underestimate the biological response to the lower intensity light levels experienced on the night shift schedule.

A limitation of the current study is the measurement of light exposure using a wrist-worn actigraph device, which is an imperfect estimate of retinal light exposure^43^. Future work using a sensor worn at eye-level, or better estimates of overall environmental lighting conditions, would provide a more accurate measure and potentially improve model estimations. Validation of models using wrist-worn sensors is important, however, given their current ubiquity and ease of use in field studies. Additionally, it is now recognized that photopic lux is limited in its ability to quantify the effect of light on the circadian system^44^. The circadian pacemaker is particularly sensitive to the phase-resetting effects of short-wavelength light^45,46^; however, the model’s light input is still based on photopic lux^18,19^. Modification of the model to account for other dimensions of light (e.g., melanopic lux^44^), along with improved light sensors, such as in^47^, would likely provide a more accurate estimate of the circadian phase response to the changed light-dark cycle in shift workers.

In conclusion, this study indicates the potential utility of a mathematical modeling approach to estimating circadian phase in a real-world shift work context, as an alternative to costly and/or burdensome measurements of circadian phase markers. The study also identified important caveats or limitations to the approach, including lower prediction accuracy across night shifts than on diurnal shifts, and failure to capture the full range of inter-individual differences, meaning while the model performs well for most individuals, the model may perform poorly in individuals with extreme early or late phase angles of entrainment. With further model development, there is potential for use of this approach to predict times of worst performance impairment, which would inform both personal and occupational countermeasures.

## Methods

### Study protocol

Participants were 20 nurses (15 female) and 5 doctors (3 female), aged 33.3 ± 9.2 y (mean ± SD) with a BMI of 23.8 ± 3.7 kg/m^2^, working rotating shifts in an Intensive Care Unit (ICU) at Austin Health, Melbourne, Australia. Doctors worked a consistent rotating roster of 7 consecutive day shifts (08:00–20:30 h) and 7 consecutive night shifts (20:00–08:30 h), with 7 days off in between each block of shifts (i.e., 7 day, 7 off, 7 night, 7 off). Nurses worked variable shift schedules consisting of day (07:00–15:30 h), evening (15:00–21:30 h), and night (20:00–08:30 h) shifts. Participants were recruited via scheduled in-service presentations and targeted recruitment during shifts. Recruitment sought workers with an upcoming roster consisting of at least 4 day or evening shifts or days off (the ‘*diurnal schedule’*) followed by a minimum of 3 consecutive night shifts (the ‘*night schedule’*).

### Study approval

All participants provided written informed consent prior to enrolment into the study, and received financial compensation for their time. The study protocol was approved by the Austin Health and Monash University Human Research Ethics Committees and was conducted in compliance with standards set by the latest revision of the Declaration of Helsinki.

### Light exposure and activity measurements

Light (photopic lux) was recorded in one-minute epochs across the entire shift pattern (diurnal schedule followed by night schedule) using a wrist actigraph device (Actiwatch Spectrum or Spectrum Plus, Philips Respironics, Bend, OR, USA; serviced and calibrated prior to data collection by Philips Respironics), worn on the non-dominant wrist. The same device also recorded wrist activity (activity counts/minute). Participants were asked to wear the device at all times, with the light sensor uncovered, except while showering or when the device would interfere with hospital operational requirements.

### Questionnaires

Daily work diaries were completed in which participants recorded the start and end times of each shift worked, including breaks. All shift times were confirmed using payroll information provided by hospital administration. Participants also completed an online sleep-health questionnaire, which included demographic information such as age, sex, body mass index (BMI), and shift work history. Diurnal preference was measured using the Horne & Ostberg Composite Morningness Eveningness Questionnaire (MEQ)^48^.

### Reference circadian phase measurements

Circadian phase was assessed by measuring the rhythm of the urinary melatonin metabolite 6-sulphatoxymelatonin (aMT6s) over 24–48 hours on both the diurnal schedule (day/evening shifts or days off) and end of the night schedule (night 3, 4 or 5). Participants collected all urine voids over the collection period, in 4-hourly sequential blocks (8-hourly during sleep). For each block the total urine volume and sampling time were recorded, and a 5-mL sample retained and stored at −20 °C. Samples were analysed for aMT6s concentration using radioimmunoassay^49^ at the Adelaide Research Assay Facility (University of Adelaide, Australia) with reagents provided by Stockgrand Ltd (University of Surrey, Guildford, UK). Assay details are reported elsewhere^11^.

Cosinor analysis was conducted to determine the acrophase (peak) time in the aMT6s rhythm^50^ at two time points: once during a sequence of confirmed diurnal schedules, and once on the final night shift. All included urine collections passed visual inspection of the aMT6s rhythm, and had a significant cosinor fit (98% had α = 0.10, 2% (n = 2) p < 0.12).

### Sleep assessment

Rest intervals were determined based on activity counts using Actiware software (Actiware 6 software, Philips Respironics, Bend, OR, USA). Main rest intervals were examined for the following criteria: minimum duration of 3 hours, mean activity below a proprietary threshold, and mean light levels below a proprietary threshold. Rest intervals that did not meet these criteria were visually inspected: time boundaries were adjusted at the start and end of each episode where there was a discrepancy between the rest interval and the activity and light distributions of ≥ 60 minutes. Rest intervals shorter than 3 hours that occurred outside the expected sleep window were marked as naps and excluded from the non-photic input into the model. Five rest intervals from four participants were missing due to the participant removing the actigraph overnight. For these cases, where off-wrist intervals aligned approximately with the time and duration of the expected sleep episode, activity and light levels were set to zero, and treated as a main rest interval.

The mid-sleep time was calculated as the mid-point between sleep onset and sleep offset for all main sleep intervals, for use in model initialization (described below). Main rest intervals only were used to calculate the square waveform non-photic input for the model (details below).

### Model structure

**Figure 6:**
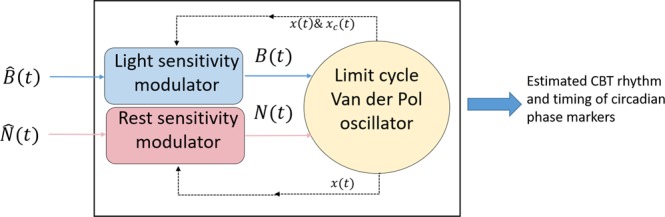
Schematic of the model of the central pacemaker driven by light via $\hat{{\rm{B}}}({\rm{t}})$ and rest derived from activity via $\hat{{\rm{N}}}({\rm{t}})$. The model is a limit-cycle oscillator of Van der Pol type in the $({\rm{X}},{{\rm{X}}}_{{\rm{c}}})$ plane (see Eqs 1 and 2). The light ${\rm{B}}({\rm{t}})$) and activity (${\rm{N}}({\rm{t}})$) driving terms are obtained from the corresponding external stimuli through specific sensitivity modulators depending on the oscillator state (see Eqs 3 and 4). $\hat{{\rm{B}}}({\rm{t}})$ is obtained from the light intensity measurements and $\hat{{\rm{N}}}({\rm{t}})$ from the activity counts. Both are collected by a wrist-worn device with a one-minute sampling rate and result from non-linear processing stages described in^19^. The output of the model consists of time-dependent trajectories in the phase plane, from which daily core body temperature minimum times are estimated.

In this study we used an existing limit-cycle oscillator model^19^ implemented with two approaches: (a) using the photic drive ${\rm{B}}({\rm{t}})$ only (photic model, ${\rm{N}}({\rm{t}})$ set to zero), and (b) using both the photic ${\rm{B}}({\rm{t}})$ and non-photic ${\rm{N}}({\rm{t}})$ driving terms (PNP model), see Fig. 6.

The main pacemaker equations are listed below, which describe the modeling framework used to calculate circadian phase estimations.

1 $$\frac{d}{dt}x(t)=\,\frac{\pi }{12}[{x}_{c}(t)+\mu (\frac{1}{3}x+\frac{4}{3}{x}^{3}-\frac{256}{105}{x}^{7})+B(t)+N(t)\,]$$

2 $$\frac{d}{dt}{x}_{c}(t)=\frac{\pi }{12}\{{L}_{q}B(t){x}_{c}(t)-x(t)\cdot [{(\frac{24}{0.99729\cdot {\tau }_{c}})}^{2}+{L}_{k}B(t)]\}$$

3 $$B(t)=\hat{B}(t)\cdot (1-{l}_{s}x(t))\cdot (1-{l}_{s}{x}_{c}(t))$$

4 $$N(t)=\,\hat{N}(t)\cdot (1-\,\tanh ({a}_{s}x(t)))$$

Equations (1) and (2) define a limit-cycle oscillator in the $\{{\rm{X}}({\rm{t}}),{{\rm{X}}}_{{\rm{c}}}({\rm{t}})\}$ state plane, via a pair of first order equations where *x*(*t*) reflects daily time variations of endogenous core body temperature, and ${x}_{c}(t)$ is a complementary variable based on a Lienard transformation. This enables the pacemaker to be modeled in a two-dimensional plane. The resulting state vector generates elliptic-like trajectories over time, the shapes of which are modulated by external stimuli. Equations (3) and (4) are circadian modulators, respectively, for the photic ${\rm{B}}({\rm{t}})$ and non-photic ${\rm{N}}({\rm{t}})$ stimuli. In Eq. (3), $\hat{B}(t)$ is the output of a dynamic light processor fed with the measurements of white light intensity (photopic lux) over successive one minute epochs. Similarly, $\hat{N}(t)$ results from the processing of activity counts from wrist actigraphy, enabling the epochs to be tagged in terms of wake or rest status. In the above equations, there are 6 additional model parameters, namely:

1. μ: stiffness parameter, set to 0.13
2. τ_*c*_: intrinsic period, set to 24.2 h
3. *L*_*q*_: model parameter, set to 1/3
4. *L*_*k*_: model parameter, set to 0.55
5. *l*_*s*_: light sensitivity parameter, set to 0.4
6. *a*_*s*_: activity sensitivity parameter, set to 10.0

All details relevant to the computation of $\hat{B}(t)$ and $\hat{N}(t)\,\,$from the light and activity measurements have been described previously^19^, including the model parameter values that have been used in the present work. All data pre-processing and phase estimations were implemented in Matlab (R2016b, Mathworks).

The model reports core body temperature minimum (CBTmin) as the circadian phase marker. Based on published relationships between CBTmin and plasma melatonin midpoint^51^, and between plasma melatonin midpoint and urinary aMT6s acrophase^52–54^, it was assumed that the timing of aMT6s acrophase in the current sample may be used as a proxy for CBTmin for the purpose of comparing model phase estimations with measured aMT6s acrophase times.

### Initialization procedure

Equations (1) and (2) require initial values {x(t_0_), x_c_(t_0_)} at the start time, t_0_, of each recording. To derive these initial values, average mid-sleep time was used as an estimate of the first CBT nadir, which is converted to an initial position in phase space, based on assuming a uniform angular velocity and constant amplitude of the rhythm. Average mid-sleep time was calculated for each individual over the diurnal shift schedule, prior to the subsequent night shift schedule. This approach relies on previously reported relationships between average mid-sleep time and circadian phase (dim light melatonin onset (DLMO)) on a regular diurnal sleep schedule^55,56^, such that mid-sleep time was used as a proxy for initial circadian phase.

To test whether having prior phase measurements could improve model predictions, an alternate initialization procedure was also tested for the night schedules, where the model was initialized using the aMT6s acrophase times measured on the diurnal schedule. We refer to this alternate initialization as the “oracle” method to emphasize that use is made of a reference acrophase time to derive initial parameter values for Eqs (1) and (2), as opposed to the applied heuristic (i.e., average mid-sleep time on diurnal schedule), which does not require a prior phase measurement.

### Data analysis

Statistical analyses were performed using SPSS version 23 (SPSS Inc., Chicago, IL, USA).

Measured aMT6s phase shift from diurnal schedule to the end of the night schedule was calculated as diurnal acrophase minus night acrophase, such that negative values indicate a phase delay and positive values indicate a phase advance. Predicted acrophase times on the diurnal and night schedules on the same dates that measurements were taken were used to calculate an estimated phase shift (i.e., predicted phase shift). Pearson’s correlations were used to examine the relationship between measured aMT6s acrophase and predicted acrophase times on both the diurnal and night schedules, and between measured and predicted phase shifts between diurnal and night schedules. Planned paired samples t-tests were used to compare estimated circadian phase to measured aMT6s phase, and to compare the prediction error between photic and PNP models at each time point. Pearson’s correlations were used to examine whether age, MEQ, BMI, and measured aMT6s acrophase were related to individual differences in prediction error on the diurnal schedule or night shift schedule, or to predicted phase shift.

## Supplementary information

Supplementary Material

## Data Availability

Materials and data in this publication can be requested via the CRC for Alertness, Safety and Productivity (Alertness CRC) by emailing inquiries@alertnesscrc.com.

## References

[1] Arendt J (2010). Shift work: coping with the biological clock. Occupational Medicine.

[2] Rajaratnam SW, Arendt J (2001). Health in a 24-h society. The Lancet.

[3] Smith MR, Eastman CI (2008). Night shift performance is improved by a compromise circadian phase position: study 3. Circadian phase after 7 night shifts with an intervening weekend off. Sleep.

[4] Czeisler CA (1999). Stability, precision, and near-24-hour period of the human circadian pacemaker. Science.

[5] Crowley SJ, Lee C, Tseng CY, Fogg LF, Eastman CI (2004). Complete or partial circadian re-entrainment improves performance, alertness, and mood during night-shift work. Sleep.

[6] Folkard S (2008). Do permanent night workers show circadian adjustment? A review based on the endogenous melatonin rhythm. Chronobiology International.

[7] Dumont M, Benhaberou-Brun D, Paquet J (2001). Profile of 24-h light exposure and circadian phase of melatonin secretion in night workers. Journal of Biological Rhythms.

[8] Gibbs M, Hampton S, Morgan L, Arendt J (2007). Predicting circadian response to abrupt phase shift: 6-sulphatoxymelatonin rhythms in rotating shift workers offshore. Journal of Biological Rhythms.

[9] Barnes RG, Deacon S, Forbes MJ, Arendt J (1998). Adaptation of the 6-sulphatoxymelatonin rhythm in shiftworkers on offshore oil installations during a 2-week 12-h night shift. Neuroscience Letters.

[10] Hansen JH, Geving IH, Reinertsen RE (2010). Adaptation rate of 6-sulfatoxymelatonin and cognitive performance in offshore fleet shift workers: a field study. International Archives of Occupational and Environmental Health.

[11] Stone JE (2018). Temporal dynamics of circadian phase shifting response to consecutive night shifts in healthcare workers: role of light-dark exposure. The Journal of Physiology.

[12] Czeisler, C. A. & Gooley, J. In *Cold Spring Harbor Symposia on Quantitative Biology*. 579–597 (Cold Spring Harbor Laboratory Press, 2007). 10.1101/sqb.2007.72.064 18419318

[13] Moore RY (1997). Circadian rhythms: basic neurobiology and clinical applications. Annual Review of Medicine.

[14] Klerman EB (2005). Clinical aspects of human circadian rhythms. Journal of Biological Rhythms.

[15] Ftouni S (2015). Ocular measures of sleepiness are increased in night shift workers undergoing a simulated night shift near the peak time of the 6-sulfatoxymelatonin rhythm. Journal of Clinical Sleep Medicine.

[16] Lamond N (2003). The impact of a week of simulated night work on sleep, circadian phase, and performance. Occupational and Environmental Medicine.

[17] Dijk D-J, Duffy JF, Czeisler CA (1992). Circadian and sleep/wake dependent aspects of subjective alertness and cognitive performance. Journal of Sleep Research.

[18] Jewett ME, Forger DB, Kronauer RE (1999). Revised limit cycle oscillator model of human circadian pacemaker. Journal of Biological Rhythms.

[19] St Hilaire MA (2007). Addition of a non-photic component to a light-based mathematical model of the human circadian pacemaker. Journal of Theoretical Biology.

[20] Boivin DB, Duffy JF, Kronauer RE, Czeisler CA (1996). Dose-response relationships for resetting of human circadian clock by light. Nature.

[21] Jewett ME, Kronauer RE, Czeisler CA (1994). Phase-amplitude resetting of the human circadian pacemaker via bright light: a further analysis. Journal of Biological Rhythms.

[22] Jewett ME (1997). Human circadian pacemaker is sensitive to light throughout subjective day without evidence of transients. American Journal of Physiology-Regulatory, Integrative and Comparative Physiology.

[23] Khalsa SB (1997). Type 0 resetting of the human circadian pacemaker to consecutive bright light pulses against a background of very dim light. Sleep Research.

[24] Rimmer DW (1995). Effectiveness of intermittent light pulses on phase-shifting of the human circadian pacemaker. Sleep Research.

[25] Kronauer RE, Forger DB, Jewett ME (1999). Quantifying human circadian pacemaker response to brief, extended, and repeated light stimuli over the phototopic range. Journal of Biological Rhythms.

[26] Aubert, X. A. In *European Biological Rhythms Society Congress* (2011).

[27] Woelders, T., Beersma, D. G. M., Gordijn, M. C. M., Hut, R. A. & Wams, E. J. Daily light exposure patterns reveal phase and period of the human circadian clock. *Journal of Biological Rhythms*, 0748730417696787 (2017). 10.1177/0748730417696787 PMC5476188 28452285

[28] Phillips AJ (2017). Irregular sleep/wake patterns are associated with poorer academic performance and delayed circadian and sleep/wake timing. Scientific Reports.

[29] Phillips AJ, Chen P, Robinson P (2010). Probing the mechanisms of chronotype using quantitative modeling. Journal of Biological Rhythms.

[30] Klerman EB, Beckett SA, Landrigan CP (2016). Applying mathematical models to predict resident physician performance and alertness on traditional and novel work schedules. BMC Medical Education.

[31] Wyatt JK, Cecco ARD, Czeisler CA, Dijk DJ (1999). Circadian temperature and melatonin rhythms, sleep, and neurobehavioral function in humans living on a 20-h day. American Journal of Physiology-Regulatory, Integrative and Comparative Physiology.

[32] Duffy JF (2011). Sex difference in the near-24-hour intrinsic period of the human circadian timing system. Proceedings of the National Academy of Sciences.

[33] Phillips, A. J. K. *et al.* High sensitivity and interindividual variability in the response of the human circadian system to evening light. *Proceedings of the National Academy of Sciences* **116**, 12019–12024 (2019). 10.1073/pnas.1901824116 PMC6575863 31138694

[34] Goel N, Lee TM (1995). Sex differences and effects of social cues on daily rhythms following phase advances in Octodon degus. Physiology & Behavior.

[35] Karatsoreos IN, Butler MP, LeSauter J, Silver R (2011). Androgens modulate structure and function of the suprachiasmatic nucleus brain clock. Endocrinology.

[36] Duffy JF, Zeitzer JM, Czeisler CA (2007). Decreased sensitivity to phase-delaying effects of moderate intensity light in older subjects. Neurobiology of Aging.

[37] Santhi N (2012). The spectral composition of evening light and individual differences in the suppression of melatonin and delay of sleep in humans. Journal of Pineal Research.

[38] Mott C, Dumont G, Boivin DB, Mollicone D (2011). Model-based human circadian phase estimation using a particle filter. IEEE Transactions on Biomedical Engineering.

[39] Brown EN, Choe Y, Luithardt H, Czeisler CA (2000). A statistical model of the human core-temperature circadian rhythm. American Journal of Physiology-Endocrinology and Metabolism.

[40] Indic P, Brown EN (2006). Characterizing the amplitude dynamics of the human core-temperature circadian rhythm using a stochastic–dynamic model. Journal of Theoretical Biology.

[41] Srinivasan, P., Dean, D. A., Beckett, S., Horowitz, T. & Klerman, E. B. Actigraphy as input to a circadian light model predicts relative impact of bright light and sleep schedule on circadian phase during a simulated shift-work protocol. *Sleep ***34**, A330–A330 (2011).

[42] Zeitzer JM, Dijk D, Kronauer RE, Brown EN, Czeisler CA (2000). Sensitivity of the human circadian pacemaker to nocturnal light: melatonin phase resetting and suppression. The Journal of Physiology.

[43] Figueiro M, Hamner R, Bierman A, Rea M (2013). Comparisons of three practical field devices used to measure personal light exposures and activity levels. Lighting Research &. Technology.

[44] Lucas RJ (2014). Measuring and using light in the melanopsin age. Trends in Neurosciences.

[45] Cajochen C (2005). High sensitivity of human melatonin, alertness, thermoregulation, and heart rate to short wavelength light. The Journal of Clinical Endocrinology & Metabolism.

[46] Lockley SW, Brainard GC, Czeisler CA (2003). High sensitivity of the human circadian melatonin rhythm to resetting by short wavelength light. The Journal of Clinical Endocrinology & Metabolism.

[47] Rabstein S (2019). Differences in twenty-four-hour profiles of blue-light exposure between day and night shifts in female medical staff. Science of The Total Environment.

[48] Shahid, A., Wilkinson, K., Marcu, S. & Shapiro, C. In *STOP, THAT and One Hundred Other Sleep Scales* 137–140 (Springer, 2011).

[49] Aldhous ME, Arendt J (1988). Radioimmunoassay for 6-sulphatoxymelatonin in urine using an iodinated tracer. Annals of Clinical Biochemistry.

[50] Nelson W, Tong L, Lee J, Halberg F (1979). Methods for cosinor-rhythmometry. Chronobiologia.

[51] Shanahan, T. L., & Czeisler, C. A. Light exposure induces equivalent phase shifts of the endogenous circadian rhythms of circulating plasma melatonin and core body temperature in men. *The Journal of Clinical Endocrinology & Metabolism ***73**, 227-235 (1991). 10.1210/jcem-73-2-227 1856258

[52] Nowak R, McMillen IC, Redman J, Short RV (1987). The correlation between serum and salivary melatonin concentrations and urinary 6‐hydroxymelatonin sulphate excretion rates: two non‐invasive techniques for monitoring human circadian rhythmicity. Clinical Endocrinology.

[53] Benloucif S (2008). Measuring melatonin in humans. Journal of Clinical Sleep Medicine.

[54] Arendt, J. *Melatonin and the mammalian pineal gland*. (Springer Science & Business Media, 1995).

[55] Martin SK, Eastman CI (2002). Sleep logs of young adults with self-selected sleep times predict the dim light melatonin onset. Chronobiology International.

[56] Kantermann T, Burgess HJ (2017). Average mid‐sleep time as a proxy for circadian phase. PsyCh Journal.

