# Supplementary material for: Application of a Limit-Cycle Oscillator Model for Prediction of Circadian Phase in Rotating Night Shift Workers
Source: Sci Rep. 2019 Jul 30;9:11032. doi: 10.1038/s41598-019-47290-6 (PMC6667480; doi:10.1038/s41598-019-47290-6)
Supplement: Supplementary file 1 — Supplementary Material [file 41598_2019_47290_MOESM1_ESM.docx]

**Title:** Application of a limit-cycle-oscillator model for prediction of circadian phase in rotating night shift workers

**Authors**: Julia E. Stone, Xavier L. Aubert, Henning Maass, Andrew J. K. Phillips, Michelle Magee, Mark E. Howard, Steven W. Lockley, Shantha M. W. Rajaratnam, Tracey L. Sletten

**Supplementary Material**

**
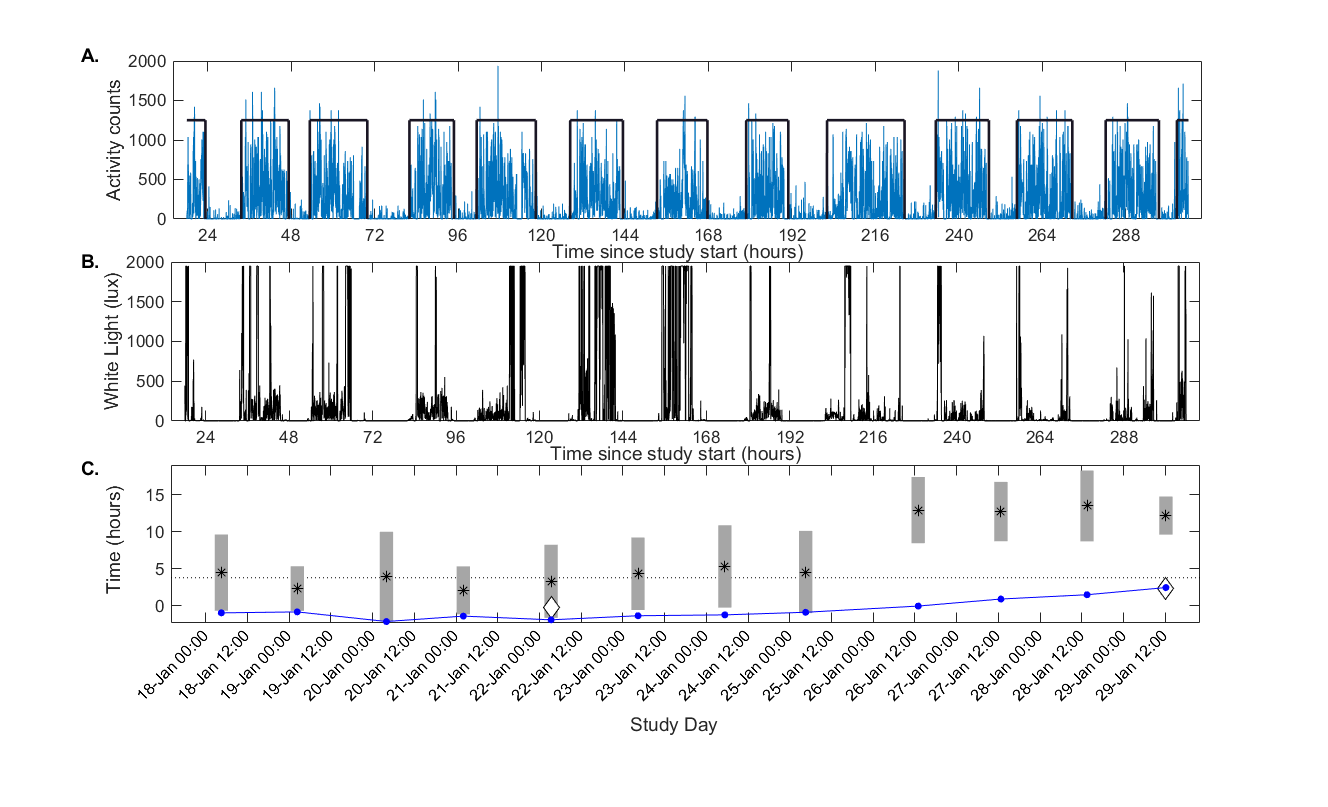
**

**Figure S1**. Example timeseries data for one participant measured over a 12 day schedule of diurnal shifts (study hours 0-216) followed by 4 consecutive night shifts (study hours 216-300). Panel A) shows raw activity data measured via wrist actigraphy (blue bars) and the calculated sleep-wake square waveform (black line) used as input into the PNP model; (B) shows pre-processed light data in lux measured via wrist actigraphy, used as the photic input; (C) shows individual rest episodes (grey bars; asterisks indicate mid-sleep time), aMT6s acrophase (diamonds) and predicted phase trajectory using the PNP model (blue circles)

**Figure S2.** Relationship between circadian phase estimated using photic model (left panel) and the PNP model (right panel), initialised using diurnal aMT6s acrophase rather than average mid-sleep time over the diurnal schedule, and measured aMT6s phase on night schedule (A and B), and aMT6s phase shift (C and D). Positive phase shift indicates a phase advance (i.e., final night shift phase occurring at an earlier clock time than day-active phase); negative phase shift indicates a phase delay (i.e., final night shift phase occurring at a later clock time than day-active phase.

**Description of light intensity comparison between shift schedules**

Light levels were compared between the diurnal and night schedules by examining light exposure as a percentage of wake time spent: below 10 lux (dim indoor light); between 10 and 100 lux (dim-moderate indoor light); between 100 and 1000 lux (moderate-bright indoor light or dim outdoor light); and ≥1000 lux (outdoor light levels; ^1^). The percentage of time spent in each light threshold on the diurnal schedule compared to the night shift schedule was examined using paired-samples t tests (Figure S2).

On average, subjects spent a greater proportion of wake time was spent between 100 and 1000 lux (t(24) = 6.93, p < 0.001) and above 1000 lux (t(24) = 5.41, p < 0.001) on the diurnal schedule compared to during the night shift schedule. Conversely, a greater proportion of time was spent in lower light levels (less than 10 lux; t(24) = -7.30, p < 0.001) and between 10 and 100 lux (t(24) = -2.19, p = 0.04)) during the night shift schedule compared to diurnal schedule.

**Figure S3.** Percentage of wake time spent in each light intensity category on the diurnal schedule (black) and night shift schedule (white). *p < 0.05, **p < 0.01 for comparisons between diurnal and night shift schedules.

**Impact of circadian phase error on estimated psychomotor performance**

Median reaction time (RT) on a psychomotor vigilance task (PVT) across the biological day, measured during a forced desynchrony protocol^2^ was used to estimate the impact of using incorrect circadian phase predictions to predict psychomotor performance. RT predicted at each circadian phase with a ± 1-hour error and ± 2-hour error, and the absolute mean difference between reported RT and that estimated with a 1-hour or 2-hour error in phase was calculated (Fig 6A). The error effect size was calculated as the mean absolute difference in milliseconds, divided by the standard deviation, at each circadian phase. Standard deviation was calculated from the reported standard error of median RT for n = 6 subjects.

The error effect size was largest at two phases (120 and 300 degrees), corresponding to the start and end of the circadian nadir in performance (Fig 6B). The error effect size is medium with a ± 1-hour error in circadian phase, and large with a ± 2-hour error in phase. This simulation illustrates that the impact of having an incorrect phase estimate is higher in terms of predicted reaction time at particular biological times, and that a larger discrepancy between predicted and actual circadian phase results in a larger error effect size at those times.

**Table S1.** Summary of prediction error in hours for photic only and PNP models across the shift schedule, with each model initialised using diurnal aMT6s phase (“oracle” model).

|  | **n** | **Predicted Phase Mean (h)** | **Predicted Phase Range (h)** | **Prediction Error Mean**  **(h)** | **Prediction Error Absolute Mean (h)** | **Prediction Error Min (h)** | **Prediction Error Max (h)** | **Prediction within ±30 min** | **Prediction within ±60 min** | **Prediction within ±120 min** |
| --- | --- | --- | --- | --- | --- | --- | --- | --- | --- | --- |
| *Night Schedule* |  |  |  |  |  |  |  |  |  |  |
| Photic Oracle Model | 25 | 4.54 (0.77) | 3.00 - 5.72 | 0.78 (1.44) | 1.20 (1.10) | 0.01 | 4.67 | 32% | 52% | 80% |
| PNP Oracle Model | 25 | 5.33 (1.11) | 3.49 - 7.74 | -0.01 (1.22) | 0.94 (0.75) | 0.00 | 3.09 | 28% | 68% | 92% |
| *Phase Shift* |  |  |  |  |  |  |  |  |  |  |
| Photic Oracle Model | 25 | -0.71 (0.75) | -2.41 - 0.70 | -0.64 (1.52) | 1.26 (1.03) | 0.01 | 3.67 | 32% | 52% | 76% |
| PNP Oracle Model | 25 | -1.70 (1.05) | -3.61 - 0.09 | 0.35 (1.32) | 1.04 (0.86) | 0.08 | 3.19 | 36% | 64% | 88% |

**Supplementary References**

1 Scheuermaier, K., Laffan, A. M. & Duffy, J. F. Light exposure patterns in healthy older and young adults. *Journal of Biological Rhythms* **25**, 113-122 (2010).

2 Wyatt, J. K., Cecco, A. R. D., Czeisler, C. A. & Dijk, D. J. Circadian temperature and melatonin rhythms, sleep, and neurobehavioral function in humans living on a 20-h day. *American Journal of Physiology-Regulatory, Integrative and Comparative Physiology* **277**, R1152-R1163 (1999).
